# Comparing joint kinematics and center of mass acceleration as feedback for control of standing balance by functional neuromuscular stimulation

**DOI:** 10.1186/1743-0003-9-25

**Published:** 2012-05-06

**Authors:** Raviraj Nataraj, Musa L Audu, Ronald J Triolo

**Affiliations:** 1 Biomedical Engineering Department, Case Western Reserve University, Cleveland, OH, USA; 2 Motion Study Laboratory, Louis Stokes Veterans Affairs Medical Center, Cleveland, OH, USA; 3 Orthopaedics Department, Case Western Reserve University, Cleveland, OH, USA

**Keywords:** Biomechanics, Standing balance, Biomedical engineering technology, Rehabilitation

## Abstract

**Background:**

The purpose of this study was to determine the comparative effectiveness of feedback control systems for maintaining standing balance based on joint kinematics or total body center of mass (COM) acceleration, and assess their clinical practicality for standing neuroprostheses after spinal cord injury (SCI).

**Methods:**

In simulation, controller performance was measured according to the upper extremity effort required to stabilize a three-dimensional model of bipedal standing against a variety of postural disturbances. Three cases were investigated: proportional-derivative control based on joint kinematics alone, COM acceleration feedback alone, and combined joint kinematics and COM acceleration feedback. Additionally, pilot data was collected during external perturbations of an individual with SCI standing with functional neuromuscular stimulation (FNS), and the resulting joint kinematics and COM acceleration data was analyzed.

**Results:**

Compared to the baseline case of maximal constant muscle excitations, the three control systems reduced the mean upper extremity loading by 51%, 43% and 56%, respectively against external force-pulse perturbations. Controller robustness was defined as the degradation in performance with increasing levels of input errors expected with clinical deployment of sensor-based feedback. At error levels typical for body-mounted inertial sensors, performance degradation due to sensor noise and placement were negligible. However, at typical tracking error levels, performance could degrade as much as 86% for joint kinematics feedback and 35% for COM acceleration feedback. Pilot data indicated that COM acceleration could be estimated with a few well-placed sensors and efficiently captures information related to movement synergies observed during perturbed bipedal standing following SCI.

**Conclusions:**

Overall, COM acceleration feedback may be a more feasible solution for control of standing with FNS given its superior robustness and small number of inputs required.

## Background

The goal of this study was to assess the potential performance of control systems employing feedback of total body center of mass (COM) acceleration and proportional-derivative joint feedback from the ankles, knees, hips, and trunk for comprehensive control of standing after spinal cord injury (SCI). Neuroprostheses employing functional neuromuscular stimulation (FNS) have been proven clinically effective for restoring basic standing function following SCI [1] using pre-programmed patterns of stimulation to produce the sit-to-stand maneuver and continuous stimulation at constant levels to maintain upright posture. Under constant stimulation, balance is maintained through upper extremity (UE) loads applied to the environment (e.g., walker, countertop). Sustained UE loading compromises the utility of standing with FNS by limiting the functional use of the hands and arms and reducing standing time due to rapid upper body fatigue. Feedback control of stimulation is necessary to provide automatic postural adjustments that reduce the UE effort necessary for stabilization.

Previous investigations into feedback control of FNS for standing after SCI have focused on servo-type joint feedback control of stimulation at the knees [2,3], hips [4,5], and ankles [6]. However, these studies restricted control actions to a single joint or anatomical plane, omitted feedback control at the trunk, and held joints not under direct feedback control in extension by constant stimulation or extensive mechanical bracing. Consequently, current standing systems still rely exclusively on continuous open-loop stimulation. The next step toward clinically acceptable closed-loop control of standing with FNS includes development of *comprehensive* feedback control systems that simultaneously coordinate actions at multiple joints to balance posture in three-dimensions (3-D).

In simulation, we have previously developed comprehensive FNS control systems utilizing either proportional-derivative joint feedback [7] or total body center of mass (COM) acceleration feedback [8] to drive an artificial neural network (ANN) trained to output changes in muscle excitation levels and maintain standing posture against disturbances. A model-based approach was employed to evaluate controller performance prior to online testing with SCI subjects. The control systems were developed and tested using a 3-D musculoskeletal model of human bipedal stance that included realistic representations of the effects of SCI on the contractile properties of stimulated muscles. A quantitative formulation for UE loading was created to interact with the standing model and simulate the stabilization forces that a user may volitionally exert on the environment. The simulated performance of the FNS control systems were mainly assessed according to minimization of the UE loading applied during postural perturbations.

In this study, the two feedback control systems were further evaluated and directly compared to outline important considerations for deployment in clinical practice. The robustness of each control system was explored in terms of the degradation in performance with systematic introduction of realistic sensor feedback errors. The two control systems were also combined (feedback = joint kinematics + COM acceleration) using a global search algorithm [9] to tune gains for all feedback inputs simultaneously and minimize UE loading when both control systems acted concurrently. Finally, systematic external force perturbations were applied to a subject with SCI while standing with constant stimulation to observe the resultant changes in joint position and linear segmental acceleration. These pilot data represented the information expected during live operation of each control system while standing with FNS in the presence of disturbances. The data were subsequently evaluated for information content and determination of the inputs necessary to accurately characterize the feedbacks required for each control system.

## Methods

### Overview of control systems developed in simulation

**Figure 1:**
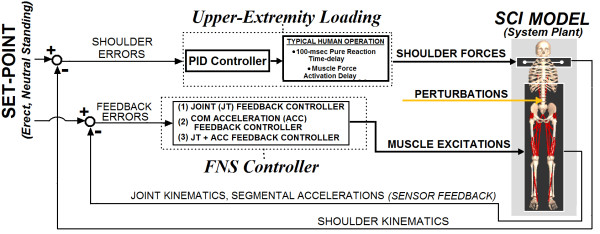
Overall Model System.

The overall model system (Figure 1) included parallel systems for FNS control and UE loading both acting to maintain the erect setpoint posture of a 3-D model of bipedal SCI stance against postural perturbations. The FNS controller modulated excitation of the paralyzed muscles across the trunk and lower extremities according to joint kinematics (JT) feedback, COM acceleration (ACC) feedback, or combined (JT + ACC) feedback. UE loading that a standing neuroprosthesis user may exert on a support device was represented as stabilization forces applied at each shoulder position to resist postural perturbations. All feedback gains for the FNS controller were optimized using a global-search algorithm to minimize UE controller output (i.e., minimize user “UE loading”) against postural perturbations.

#### Three-dimensional model of bipedal standing

The 3-D musculoskeletal model of human bipedal stance was adapted from a previously described representation of the lower extremities [10] and trunk [11]. The model consisted of nine segments (two feet, two thighs, two shanks, pelvis-lumbar component, and head-arm-trunk complex) with 15 anatomical degrees of freedom (DOFs) representing bilateral motions of ankle plantar/dorsiflexion (PF/DF), ankle inversion/eversion (Inv/Ev), knee flexion/extension (F/E), hip F/E, hip internal/external rotation (Int/Ext), and hip ab/adduction (Ab/Ad). These muscle groups were consistent with those targeted by an existing 16-channel implantable stimulator [12] utilized for standing. Excitation to a muscle group was a normalized (0 to 1) command input analogous to stimulation level. Muscles were represented as Hill-type actuators with nonlinear force dynamics that included excitation-activation coupling and conventional length-tension and force-velocity properties [13]. The peak force parameters for the muscle groups were scaled to produce the maximal isometric joint moments reported for individuals with complete thoracic-level SCI in response to electrical stimulation [14].

#### Baseline constant muscle excitation sets

To provide a comparative baseline for controller performance, the “optimal” and “maximal” constant excitation sets were determined for the desired setpoint posture using the optimizer from [15]. The, “optimal” excitations (CONST:OPT) represented the *minimum* constant excitation levels sufficient to support stable standing, while the “maximal” excitation (CONST:MAX) represented the *largest* constant excitation levels capable of supporting the same posture and is consistent with the approach taken clinically. Additional details about these muscle excitation sets are presented in [7].

#### Upper extremity loading

UE loads were represented as stabilization forces applied to each shoulder in three dimensions as determined by the output of a proportional-integral-derivative (PID) controller based on shoulder position errors relative to the reference setpoint posture. Standing performance was defined in terms of the total UE loading required to stabilize the model against postural disturbances. Total UE loading was calculated as the absolute net vector force applied in 3-D at each shoulder position. Forces were applied bilaterally for two-arm support conditions, and unilaterally as required for functional reaching on the contralateral side. Additional details about this model formulation for UE loading are presented in [7].

#### Joint feedback for FNS control

This control system consisted of proportional and derivative (PD) angular inputs from 9 joint DOFs: trunk pitch and bilateral ankle PF/DF, knee F/E, hip F/E, and hip Ab/Ad. Standard PD-feedback error control laws were used to drive corresponding inputs to an ANN as follows:

(1) $${\text{ANNInput}}_{i}=K_{p,i}\times \theta_{i}+K_{D,i}\times \overset{\cdot }{\theta_{i}}$$

As with standard PD control, each joint input (‘*i*’) to the ANN was the sum of the joint angle position and velocity errors (*θ’s*) multiplied by proportional (*K*_*P*_) and derivative (*K*_*D*_) gains, respectively. The errors were computed with respect to quiet standing at the neutral setpoint position. The ANN was trained to output changes in muscle excitation consistent with the maximum stiffness values achievable for typical standing by FNS following SCI. Further details outlining development and evaluation of this control system can be found in [7].

#### COM acceleration feedback for FNS control

This control system consisted of proportional feedback of anterior-posterior (AP) and medial-lateral (ML) components of total body COM acceleration to drive corresponding inputs of an ANN as follows:

(2) $$\text{ANN}\;\text{Inpu}t_{i}=K_{p,i}\times ACC_{COM-i}$$

Each acceleration component input ‘*i*’ was simply the respective measured change in COM acceleration (*ACC*_*COM*_) multiplied by the corresponding proportional gain (*K*_*P*_). The ANN was trained to output optimal changes in muscle excitation required to induce a desired net change in COM acceleration to counter the effects of postural disturbances. Further details outlining development and evaluation of this control system can be found in [8].

#### External force-pulse perturbations

A total of 978 external perturbation simulations were specified to evaluate operation of the feedback control systems. Each simulation consisted of a single load-pulse perturbation applied at a single location on the musculoskeletal model, which was initially placed at the desired erect setpoint. For assessing standing performance, UE loading was tracked during application of the perturbation and subsequent 500 msec recovery period. Perturbations were applied at the COM location of the thorax, pelvis, femur, shank, or total body in the forward, backward, left, or right directions relative to a globally fixed reference frame. These force disturbances ranged from 5% to 15% body-weight (BW) in amplitude and 50 to 500 msec in duration. Additional details of these particular simulations can be found in [7].

#### Functional task performance (FTP)

Functional implications of the controller were assessed in simulation with application of continuously varying force loads at one shoulder to mimic postural disturbances due to weighted, voluntary single arm reaching [16]. Three-dimensional, sinusoidal force loading was applied at the right shoulder while UE control was applied only at the left shoulder. The parameters of the sinusoidal forces applied were originally reported in [7]. This test was conducted over 10 seconds of simulation time with sinusoidal loading applied immediately at time = 0. However, the model did not achieve “steady-state”, i.e., COM position was less than 2 cm from the running mean, until time = 7 seconds.

#### Optimal tuning of control systems

Gains for each feedback control system were determined to minimize an objective function representing the total two-arm UE loading necessary for stabilization during perturbation and recovery over an entire set of simulated disturbances. Total two-arm UE loading was defined as the sum of the absolute net stabilization forces applied at the shoulders. The gains for every feedback input were optimally tuned using the *asynchronous parallel pattern set global search* algorithm implemented in the APPSPACK [9] software package running on a *FUSION A8* multi-processor computer (*Western Scientific, Inc*., San Diego CA). Particular algorithm parameters, tuning procedures, and resultant gain values are originally described in [7,8].

### Assessing controller robustness to expected measurement errors

To test the theoretical robustness of joint and COM acceleration feedback control, systematic errors were introduced to the feedback inputs during simulated external perturbation. Three types of typical sensor error were investigated: (1) noise, (2) dynamic tracking, and (3) placement misalignment. Performance degradation was assessed as the change in total UE loading across all simulations without feedback error (*UE*_*Controller*_) compared to feedback control in the presence of feedback error (*UE*_*Controller+Error*_). This comparison was normalized to the case of maximal continuous excitation (*UE*_*Baseline*_), which represents the clinical standard. The normalized linear relative performance index (RPI) was calculated as:

(3) $$\text{RPI}=\frac{UE_{\text{Controller}+\text{Error}}-UE_{\text{Controller}}}{UE_{\text{Baseline}}-UE_{\text{Controller}}}$$

RPI equal to 1 serves as the “equal performance boundary” where the controller with feedback error is equivalent baseline (i.e., all advantages of controller feedback effectively lost and performance degradation is 100%). For each of the following error types, simulations were run such that feedback error at the prescribed level was introduced to all feedback inputs simultaneously. Several error levels were tested including those surpassing the equal performance boundary (i.e., RPI = 1). A 3^rd^ order polynomial was fit for each error type to interpolate the error level at which RPI = 1.

#### Noise error

Sensor noise was simulated by adding a randomly generated error value for each feedback input at each time integration step. The random error value was allowed to be either positive or negative but the absolute value was required to be less than a prescribed maximal error limit.

#### Tracking error

To simulate continuous tracking error, sinusoidal error with prescribed root mean square (RMS) amplitude was added to each joint input. Since this error type occurs under dynamic conditions, the error sinusoids were applied only when angular velocity ≠ 0 for each joint or acceleration ≠ 0 for each segment.

#### Placement error

Placement error was neglected for JT control since inertial sensors capable of global orientation measurements [17] could calibrate for initial placement errors. For ACC control, accelerometers must be properly aligned according to desired anatomical reference frames (i.e., along defined AP and ML axes). Placement error was simulated by misaligning the global reference frame from which accelerations were measured by angles ranging from 0 to 90° in both positive and negative directions.

### Evaluating joint kinematics and COM acceleration from pilot data of FNS standing against systematic postural perturbations

**Figure 2:**
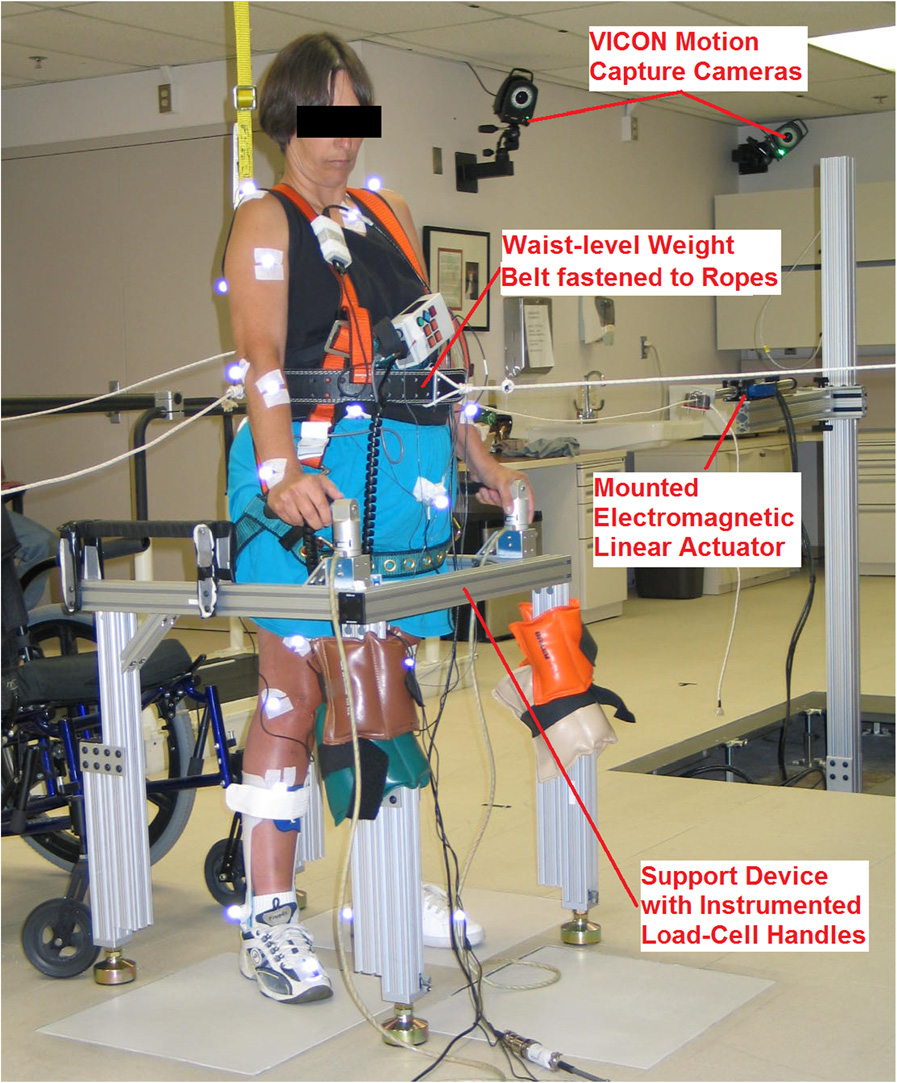
Laboratory set-up for applying external force-pulse perturbations to subject with complete T4-level spinal cord injury standing with continuous stimulation.

To generate the pilot data required to evaluate the potential signals for feedback control, systematic postural disturbances were applied to a female subject with complete thoracic-level (T4) SCI standing with continuous open-loop FNS (Figure 2) delivered by an implanted pulse generator [12]. All experiments were approved by the institutional review board of the Louis Stokes Cleveland Department of Veterans Affairs Medical Center. Stimulation was applied via intramuscular electrodes to activate sixteen muscle groups similar to those targeted by our model-based control systems. They include bilateral tibialis anterior, gastrocnemius, vasti, semimembranosus, gluteus maximus, gluteus medius, posterior adductor magnus, and erector spinae. Maximum stimulation pulse parameters (20 mA amplitude, 250 μsec duration) were applied to all muscle groups with few exceptions in order to facilitate the ability to comfortably stand erect.

External force-pulse disturbances (250 msec duration) were applied to the subject in the front, back, right, or left directions along the anatomical transverse plane approximately at the subject COM. Force-pulses were generated by four electromagnetic linear actuators (STA2506 model, *Copley Controls, Inc.,* Canton, MA) positioned orthogonally about the subject and connected to a weight-belt by nylon ropes. Force-pulses of various amplitudes based on subject tolerance were repeated 15 times in each direction across multiple sessions. Amplitude levels in the forward, backward, and lateral directions ranged from 1-9% body-weight (BW), 1-5% BW, and 2-12% BW, respectively. The subject stabilized herself by applying corrective forces with her upper extremities upon a custom instrumented support device. Additional details for the equipment, including precautions for fall prevention are described in [18].

A VICON® motion capture system (*Vicon Motion Systems and Peak Performance, Inc*., Oxford, UK) tracked the 3-D positions (at 100 Hz) of retro-reflective markers placed on 27 anatomical landmarks across the torso and upper and lower extremities as specified by the *PlugInGait* marker set (C7, clavicle, sacrum and bilaterally on shoulder, upper arm, elbow, forearm, wrist, anterior superior iliac spine, thigh, knee, tibia, ankle, heel, and toe). These marker positions were used to estimate segment COM positions according to segment definitions from [19] in order to calculate total body COM position. All position data were double-differentiated off-line and smoothed with a low-pass digital filter [20] to obtain 3-D acceleration data. These kinematic data were subsequently analyzed by principal component analysis and linear regression models.

## Results

### Controller gain tuning

The optimal controller gains determined by the global search algorithm for minimizing UE loading against external force-pulse perturbations are listed in Table 1 for all three controller cases. For joint feedback, both K_P_ and K_D_ at the knees remained at zero whether JT or JT + ACC feedback control was active. This indicates that the knees effectively remained in full extension, as normally observed during standing [21,22]. Most K_P_ and K_D_ values changed marginally (< 11%) with JT + ACC control. The major exceptions were increases (>60%) for hip Ab/Ad and the K_D_ for trunk F/E, which suggests these inputs produced synergistic effects with those of ACC. The feedback gains (K_A_) for the ACC feedback inputs were also notably re-distributed as the absolute value of K_A-ML_ increased 70% and K_A-AP_ decreased 80%. This suggests that JT more effectively stabilized in the AP dimension while ACC contributed uniquely to stability in the ML dimension. 

**Table 1:** Controller gain optimization results

**Joint feedback input**	**JOINT (JT) FEEDBACK ALONE**		**JT + ACC COMBINED**	
	**Proportionaljoint gainK**_**P**_**(unitless)**	**Derivativejoint gainK**_**D**_**(sec)**	**Proportionaljoint gainK**_**P**_**(unitless)**	**Derivativejoint gainK**_**D**_**(sec)**
Ankle PF/DF	6.49	0.52	7.17	0.51
Knee F/E	0.00	0.00	0.00	0.00
Hip Ab/Ad	2.22	1.71	3.64	3.33
Hip F/E	0.00	1.08	0.00	1.68
Trunk F/E	7.78	13.64	7.28	19.50

**Center of Mass (COM) Acceleration Dimensional Feedback Input**	**COM ACCELERATION (ACC) FEEDBACK ALONE**		**JT + ACC COMBINED**	
	**Proportional Acceleration Gain K**_**A**_**(unitless)**		**Proportional Acceleration Gain K**_**A**_** (unitless)**	
Anterior-Posterior	-5.17e-2		-1.08e-2	
Medial-Lateral	-0.992		-1.68	

### Controller performance

#### External perturbations – sample individual simulations

**Figure 3:**
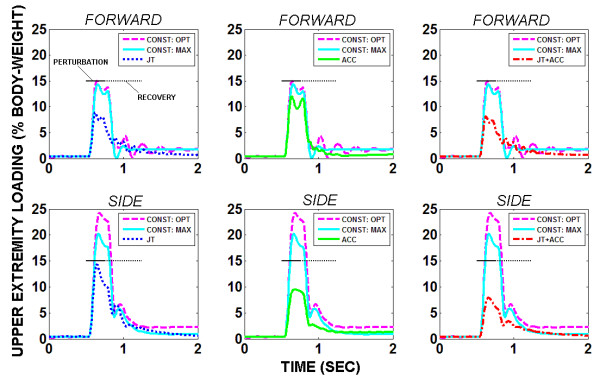
Two-arm UE loading to stabilize against perturbation pulse (15% body-weight, 250 msec) applied near model COM in either forward or side (i.e., right) direction under either optimal (CONST: OPT) or maximal constant (CONST: MAX) muscle excitation or controller modulation of muscle excitation under joint (JT), COM-acceleration (ACC) or combined (JT + ACC) feedback.

Figure 3 shows UE loading results for all five test cases for a 250 msec, 15% BW force-pulse perturbation applied near the total body COM in either the forward or side directions. Controller feedback produced stable UE loading responses whereby oscillations were dampened and steady-state achieved. Controller feedback reduced both the maximum and mean UE loading compared to either baseline case. JT produced greater reductions in UE loading than ACC against the forward disturbance while ACC outperformed JT against the sideward disturbance. Combined JT + ACC feedback demonstrated positive features of both control systems by producing greater reductions than either isolated feedback control system for both disturbance directions.

#### Perturbations – aggregate results

Table 2 denotes summary results for the mean UE loading observed in response to postural perturbations across all sub-case variables (direction, location, amplitude, duration, UE support conditions, and during simulated FTP). Using MANOVA, UE loading was significantly less using any of the feedback control cases compared to any of the baseline cases with rejection of the null hypothesis of equal means at p = 0.05 across all perturbation variables. Compared to the maximal baseline, UE loading was reduced by 43%, 51%, and 56% with ACC, JT, and JT + ACC feedback, respectively against external perturbations. During steady-state FTP, the mean reductions were 39%, 70%, and 71%, respectively.

**Table 2:** Aggregate simulation results during stabilization against postural disturbances for 5 muscle excitation test cases

	**CONST:OPT**	**CONST: MAX**	**JT Control**	**ACC Control**	**JT + ACC Control**
**Mean muscle excitation level**	**0.521**	**0.908**	**0.623**	**0.564**	**0.664**
**PERFORMANCE RESULTS: MEAN UPPER EXTREMITY LOADING (Newtons)**					
***Perturbation Direction***					
*Forward*	*66*	*47*	*19 (-60%)*	*27 (-44%)*	*18 (-62%)*
*Backward*	*32*	*22*	*17 (-23%)*	*25 (+14%)*	*17 (-23%)*
*Side (Left or Right)*	*51*	*36*	*16 (-54%)*	*14 (-61%)*	*13 (-63%)*
***Perturbation Location (Segment)***					
Thorax	61	48	31 *(-35%)*	34 *(-27%)*	30 *(-36%)*
Pelvis	58	41	22 *(-47%)*	20 *(-49%)*	19 *(-53%)*
Thigh (Left or Right)	52	35	16 *(-54%)*	19 *(-44%)*	14 *(-61%)*
Shank (Left or Right)	39	22	6 *(-72%)*	8 *(-63%)*	5 *(-77%)*
Total Body COM	70	52	27 *(-48%)*	31 *(-40%)*	23 *(-55%)*
***Support Conditions***					
Two-Arm Support	32	28	19 *(-32%)*	20 *(-29%)*	17 *(-39%)*
One-Arm Support	73	44	16 *(-64%)*	21 *(-52%)*	15 *(-66%)*
One-arm FTP*	11	11	3 *(-70%)*	7 *(-39%)*	3 *(-71%)*
Overall Upper Extremity Loading (N)**	**52.7 ± 25**	**35.9 ± 8**	**17.6 ± 3*(-51%)***	**20.7 ± 6*(-43%)***	**15.3 ± 3*(-56%)***
**KINEMATICS RESULTS (During One-Arm Support)**					
COM Position (cm)	7.3 ± 5.6	4.5 ± 1.3	1.8 ± 0.4	3.4 ± 1.6	1.8 ± 0.4
COM Acceleration (cm/sec^2^)	37 ± 15	21 ± 4	25 ± 4	20 ± 4	24 ± 4
Joint Position Error (deg)	1.3 ± 0.9	0.83 ± 0.2	0.27 ± 0.05	0.55 ± 0.2	0.25 ± 0.04
Joint Velocity Error (deg/sec)	3.0 ± 1.0	2.0 ± 0.6	1.2 ± 0.1	2.1 ± 0.7	1.3 ± 0.2

CONST: OPT = Constant Muscle Excitations, Optimal Levels; CONST: MAX = Constant Muscle Excitations, Maximal Levels; JT = Joint Feedback Control of Excitation Levels; ACC = Center of Mass Acceleration Feedback Control of Excitation Levels; JT + ACC = Combined Feedback Control of Excitation Levels.

Notes: *(X%)* = %Change from CONST:MAX case.

*Mean UE loading calculated for zeroed RMS only during “steady-state” portion of FTP.

**Overall values based on average of UE loading against discrete perturbations across two-arm and one-arm support conditions (i.e., does not include FTP) taken to additional significant digit.

Across perturbation direction, UE loading was greatest when stabilizing against forward perturbations since the standing system is inherently more unstable in the AP than ML dimension given the smaller base of support (BOS) along that direction [19]. The increase over backward perturbations was due to the setpoint posture COM being located in the center of the BOS. At this position, there was a slight forward lean allowing gravity to assist resistance against backward perturbations. The reduction in UE loading with feedback control was smallest for backward disturbances due to a lack of muscles targeted for excitation that would produce strong anterior counter moves (e.g., trunk and hip flexion). This results from limitations in the number of available stimulation channels with current implants and the clinical necessity to target extension muscle groups to provide adequately erect standing posture against gravity.

Across location, UE loading decreased as the perturbations were applied more inferiorly due to the attenuating effects of the mass-inertia of the more superior segments. With single-arm support, UE loading was significantly greater under constant muscle excitation. However, with feedback control the mean UE loading was similar with either single or double-arm support. Feedback control also consistently resulted in mean muscle excitation levels intermediate to the two baseline cases, while producing smaller changes in total body COM. As expected, feedback control of a particular variable resulted in the smallest mean changes observed for that variable (i.e., ACC → lowest mean change in COM acceleration, JT → lowest mean change in joint position and velocity errors).

For feedback control, the standard deviations were notably smaller than baseline, indicating that controller operation produced a more consistent and stable disturbance response compared to optimal constant excitation. Across either single-arm or double-arm support, UE loading results were significantly different for any controller case compared to either baseline case (p = 0.01). Using MANOVA, significant differences in UE loading for each controller case against each baseline case were also noted with rejection of the null hypothesis of equal means at p = 0.05 across all perturbation variables (direction, location, amplitude, duration). However, MANOVA was unable to demonstrate significant differences between the three controller cases, suggesting each feedback controller case has similar effectiveness.

### Controller robustness

Table 3 reports the equal performance error levels and the typical error levels for inertial sensors along with the corresponding expected degradation in performance for all error types tested. The typical error levels listed for noise or tracking were based on reported values for integrated 3-D inertial sensors [23] that could be incorporated into a physical realization of an FNS control system. Typical placement error was determined according to observed FNS standing performance against disturbances in simulation. In all test cases, the polynomial fits estimated degradation in performance at the typical sensor error level to be under 100%. This suggests an improvement in standing performance is achievable with controller feedback over constant excitation using commercially-available inertial sensors. Performance degradation due to typical tracking error was notably greater than either typical noise or placement errors, which were all < 5%. For typical tracking error, performance degradation for ACC (35%) was markedly less than JT (86%). This suggests that the margin of error in deploying JT using current sensor technology may be considerably smaller than ACC. 

**Table 3:** Controller robustness to feedback error

**Error type**	**Equal performance Error level (RPI = 1)**	**Typical sensor Error level**	**% Degradation in Controller performance at “Typical sensor error level”**
Joint Position Noise	1.01 deg	0.05 deg*	4.9
Joint Velocity Noise	6.86 deg/sec	0.05 deg/sec*	0.7
Joint Position Tracking	3.01 deg	2.60 deg*	86
Acceleration Noise	0.09 m/sec^2^	0.002 m/sec^2^*	0.6
Acceleration Tracking	0.62 m/sec^2^	0.27 m/sec^2^*	35
Accelerometer Placement	37 deg	3.2 deg max** 1.7 deg mean**	1.9 max 0.7 mean

*values reported as typical for inertial sensors used in [23].

**values based on overall mean and average maximum observed during stabilization period for computer simulations of perturbed FNS standing.

### Evaluations of pilot data from perturbed FNS standing

Using the *Statistics Toolbox* by MATLAB® (*Mathworks, Inc*., Natick, MA), principal component analysis and stepwise linear regressions were applied on the marker data collected during perturbed standing by FNS. For the position and acceleration data of all 27 markers (3-D yields a total of 81 dimensions in each data set), only 10 principal components (PCs) were required to explain over 90% of the variance in each set. This suggests that the standing SCI subject demonstrated notably simpler synergies when undergoing postural perturbations than those potentially described by 3-D position of all 27 anatomical landmarks. For the joint angle (9 position DOFs) and COM acceleration (2 dimensional components) data sets calculated as potential controller feedback for JT and ACC, respectively, 6 and 2 PCs were needed to explain over 90% of the variance in each set. For JT feedback data, the reduction in the number of evident independent features is likely due to both the simplified synergy during perturbed standing and closed-chain effects coupling changes in joint positions across the lower extremities. While 2 PCs were still required for ACC, it is still considerably less than the 6 PCs for JT.

**Figure 4:**
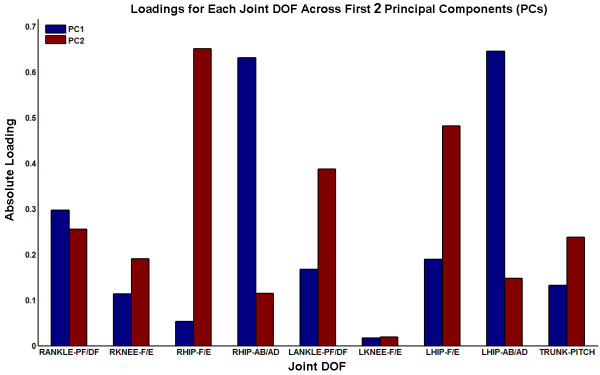
Barplot of absolute loading coefficients across first two principal components for the angular position data of the specific joint degrees of freedom (DOFs) potentially targeted by a feedback control system for FNS standing.

Figure 4 displays the individual loading coefficients for each of the joint angle DOFs targeted for feedback across the first 2 PCs. Each of the hip DOFs was loaded more upon one PC over the other. Both hip abduction/adduction DOFs had notably higher loadings on the first PC and both hip flexion/extension DOFs on the second PC. This indicates that the first PC represents more of a ML motion synergy and the second PC more of an AP synergy. Correspondingly, trunk pitch, knee flexion/extension, and left ankle plantar/dorsiflexion have relatively higher loadings on the second PC. However, right ankle plantar/dorsiflexion had higher loading on the first PC. This may be due to the right foot being generally placed in a slightly externally rotated position to accommodate postural comfort of the SCI subject who has reported joint tightness in the right ankle. The relatively smaller loading coefficients for the knee joint DOFs across both of the first two PCs is consistent with the knees being maintained in extension and not being evidently correlated with the primary motion features.

**Figure 5:**
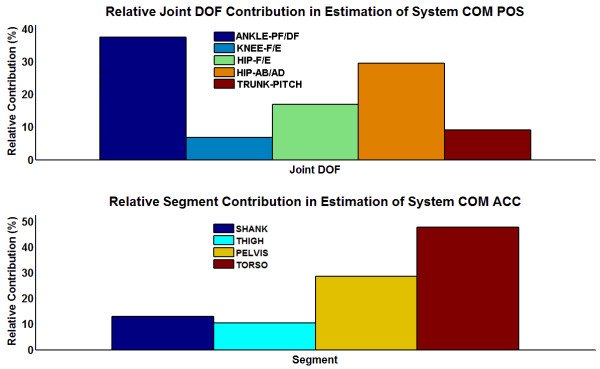
**TOP: Relative contributions by individual joint degrees of freedom (DOFs) in linear regression estimate of COM position (POS) according to absolute values of regression coefficients.** BOTTOM: Relative contributions by individual segment accelerations in linear regression estimate of COM acceleration (ACC) according to absolute values of regression coefficients. *Note*: Bilateral DOFs or segment contributions were combined.

Stepwise regression was applied to determine which joint angle positions and segmental accelerations contributed most to accurately predicting the 2-D (AP, ML) components of standing COM position and COM acceleration, respectively. All marker data were used to compute COM kinematics serving as regression model outputs and indicators of *global* system kinematics. The 9 joint position DOFs proposed for JT and 2-D accelerations of 6 major segments (pelvis, torso, both thighs, both shanks) across the trunk and lower extremities were the respective model inputs. COM position and acceleration could be estimated to explain over 90% and 99% the variance in each output data set, respectively. The stepwise linear regression analyses suggested that all 9 joint inputs and all 6 segment accelerations contributed positively (p = 0.05) in estimating 2-D COM position and COM acceleration, respectively. However, the absolute values of the individual coefficients multiplying corresponding feedback inputs for the regression model indicate the relative contributions in COM estimation as depicted in Figure 5. Clearly, ankle PF/DF and hip Ab/Ad are prime inputs as their effects contributed to over 65% of the estimate of COM position. For estimation of COM acceleration, the 2-D acceleration effects of the torso and pelvis segments contributed to over 75% of the estimate of COM acceleration.

An additional linear regression estimate was also performed using a select subset of the inputs according to fraction contributions observed in Figure 5. For these regression estimators, ankle PF/DF and hip Ab/Ad were preserved to estimate COM position and pelvis and torso accelerations were preserved to estimate COM acceleration. Using the reduced input sets, components of COM position were estimated with over 83% of the variance explained, and components of COM acceleration were estimated with still over 99% of the variance explained.

## Discussion

This study examined the potential efficacy of using either COM acceleration or joint kinematics for feedback control of comprehensive, 3-D standing by FNS following SCI. In simulation, controller performance was primarily assessed according to the reduction in UE loading to stabilize against postural disturbances that could be achieved compared to the clinical analog of maximal constant excitation of paralyzed musculature. Both JT and ACC feedback control modes significantly reduced UE loading in isolation (51%, 43%, respectively) and in combination (56%) compared to CONST:MAX against discrete external perturbations. Since JT control more comprehensively describes system kinematic states, it was expected that the greatest reduction in UE loading was observed in the presence of JT. However, isolated ACC produced over 80% the reduction as JT. This suggests that ACC alone can still largely recruit the functional capacity of the paralyzed musculature targeted for standing control by FNS. In fact, results for optimal feedback gains and performance both suggest that ACC was more effective in activating postural responses for disturbances applied laterally. JT did further outperform (70% versus 39%) ACC during FTP simulated as continuous sinusoidal loading. But activities of daily living may more typically entail individual functional reaches and thereby be better represented by discrete loads. For example, individual reaches may involve indiscriminate pauses between reaches and variable strategies in incurring the loading depending on the shape and weight of objects being manipulated.

Error analyses suggested that standing performance with ACC would be considerably more robust than JT at feedback error levels expected to be typical for this application. While JT and ACC feedback control were both robust to typical respective noise or placement sources of sensor measurement error, ACC was significantly more robust against potential tracking errors that are typical of inertial measurement sensors. This may be a product of ACC employing only two feedback inputs (AP, ML dimensions of COM acceleration) compared to eighteen JT feedback inputs (proportional, derivative values at nine joints). This is an important advantage for ACC feedback for feasible clinical deployment. Not only do fewer measured inputs reduce the likely influence of measurement contamination on controller feedback, fewer feedback gains would need to be readily tuned according to optimal performance observed under live conditions. Fewer feedback inputs may also result in a system with greater long-term clinical acceptance by requiring fewer worn sensors.

The inherent advantages in ACC over JT likely derive from the ability to describe standing by FNS against systematic postural disturbances as simpler motion synergies whereby comprehensive description of system kinematics may not be necessary for effective control. PCA demonstrated significant reductions in the number of independent linear features that exist compared to all of those measured by all postural marker data collected. This indicates that a concise set of feedback measurements may be sufficient for comprehensive postural control. Furthermore, although the JT feedback data set was composed of more dimensions than that of ACC, it did not estimate respective global (i.e., COM) kinematics as effectively.

Isolated COM acceleration feedback does theoretically preclude “hands free” standing since a measure of positional and velocity based adjustments must likely be made by the upper extremities to stabilize against postural disturbances. But this is offset by the clinical advantages in deployment of its operation relying on fewer inputs and likely fewer sensors. To this end, regression analysis of the pilot perturbation data in this study demonstrated that, potentially, only accelerometer measurements at the pelvis and torso would be necessary to accurately characterize feedback required for ACC. However, given the potential in improving performance with the addition of JT, feedback from only select joints may still be beneficial provided that it notably improves performance without significantly compromising robustness to measurement error, increasing the effort to tune the system, or adding copious amounts of worn instrumentation. To this end, regression analysis from the pilot data in this study indicates that, for sensor deployment of JT, considerations should focus on providing reliable estimation of ankle PF/DF and hip Ab/Ad. Further investigation could be performed to determine an optimal combination of joint and COM acceleration inputs that offers significant improvement in performance but requires a minimal number of sensors. However, this study suggests that ACC in isolation potentially offers a more viable clinical solution than JT for initial laboratory deployment.

While the aforementioned characteristics of ACC potentially offer improved controller performance, they also provide practical advantages in conducting future studies with live SCI individuals. Minimizing the number of sensors streamlines experimental set-ups, which can maximize data collection time in the laboratory. This can be crucial since subjects require sufficient rest between trials and may have limited availability to participate in experiments. Furthermore, sufficient pilot data needs to be collected to develop either JT or ACC control systems. This includes data to train the ANN, which establishes the relationship between changes in system kinematics due to changes in stimulation, and data used to robustly estimate COM ACC for each specific subject. Initially, a few sessions in the laboratory may be required to determine mean system characteristics. For practical clinical implementation, it would also be important to develop adaptive algorithms that autonomously tune feedback gains and optimize standing performance given time-varying muscle properties observed with prolonged system usage. This should mitigate the need of system users to return to the laboratory for frequent, comprehensive system re-tuning.

## Conclusions

In conclusion, a simulated control system that comprehensively employs proportional-derivative joint feedback and COM acceleration feedback yielded the best performance for stabilization against postural disturbances during simulated bipedal standing with FNS compared to maximal constant muscle excitation. However, this was not a significant improvement over control systems employing either joint or COM acceleration feedback alone. Furthermore, simulation results and pilot standing perturbation data collected with a SCI subject indicate that COM acceleration feedback offers numerous advantages for clinical deployment. They include providing comparable performance to comprehensive joint feedback control, requiring significantly fewer feedback inputs, and potentially robust estimation of those inputs utilizing only two sensors.

## Authors' contributions

RN conceived and designed the study, drafted the manuscript, collected and analyzed data from all simulation and laboratory experiments. MLA developed the simulation environment utilized in this study and revised the manuscript critically for intellectual content. RJT coordinated the design study and revised the manuscript critically for intellectual content. RJT also acquired funding and directed the research group. All authors read and approved the final manuscript.

## Authors’ background information

RN recently completed his Ph.D. in the Biomedical Engineering Department at Case Western Reserve University (CWRU) in Cleveland, OH, USA. His research investigated feedback control of standing balance by functional neuromuscular stimulation following spinal cord injury and was conducted as part of the Cleveland Functional Electrical Stimulation (FES) Center. MLA is a Principal Researcher with the Cleveland FES Center and Biomedical Engineering Department at CWRU. RJT is a Professor with the Orthopedics Department at CWRU, Principal Investigator at the Cleveland FES Center and Advanced Platform Technology Center, and Director of the Motion Study Laboratory at the Louis Stokes Cleveland Veterans Affairs Medical Center.
